# Efficient estimation of the total number of acini in adult rat lung

**DOI:** 10.14814/phy2.12063

**Published:** 2014-07-04

**Authors:** Sébastien F. Barré, David Haberthür, Marco Stampanoni, Johannes C. Schittny

**Affiliations:** 1 Institute of Anatomy, University of Bern, Bern, Switzerland; 2 Swiss Light Source, Paul Scherrer Institute, Villigen, Switzerland; 3 Institute for Biomedical Engineering, Swiss Federal Institute of Technology, Zürich, Switzerland

**Keywords:** Lung, pulmonary acinus, stereological analysis, X‐ray microcomputed tomography, X‐ray tomographic microscopy

## Abstract

Pulmonary airways are subdivided into conducting and gas‐exchanging airways. An acinus is defined as the small tree of gas‐exchanging airways, which is fed by the most distal purely conducting airway. Until now a dissector of five consecutive sections or airway casts were used to count acini. We developed a faster method to estimate the number of acini in young adult rats. Right middle lung lobes were critical point dried or paraffin embedded after heavy metal staining and imaged by X‐ray micro‐CT or synchrotron radiation‐based X‐rays tomographic microscopy. The entrances of the acini were counted in three‐dimensional (3D) stacks of images by scrolling through them and using morphological criteria (airway wall thickness and appearance of alveoli). Segmentation stopper were placed at the acinar entrances for 3D visualizations of the conducting airways. We observed that acinar airways start at various generations and that one transitional bronchiole may serve more than one acinus. A mean of 5612 (±547) acini per lung and a mean airspace volume of 0.907 (±0.108) *μ*L per acinus were estimated. In 60‐day‐old rats neither the number of acini nor the mean acinar volume did correlate with the body weight or the lung volume.

## Abstract (precis)

An efficient method to estimate the number of acini in young adult rats has been developed. All entrances of the acini were counted, labeled, and visualized in three‐dimensional stacks of X‐ray tomographic images. We observed that acinar airways start at various generations and that one transitional bronchiole may serve more than one acinus. A mean of 5612 (±547) acini per lung and a mean acinar airspace volume of 0.907 (±0.108) *µ*L were estimated.

## Introduction

Lungs are composed of two distinct types of airways: conducting and gas‐exchanging airways. Purely conducting airways transport the inhaled air from the trachea to the gas‐exchanging regions through a tubular system of bronchi and bronchiole. The airways distal of the conducting ones possess alveoli where the O_2_/CO_2_ gas exchange occurs at the air–blood barrier. Weibel's model of the bronchial tree (Weibel 1963) describes the human lung structure as a tree where each branch divides dichotomously into two smaller branches forming a new generation of airways. The conducting airways include approximately 10 generations of bronchi, starting at generation zero with the trachea, followed by four generations of bronchioles. The gas‐exchanging region is connected to the conducting airways and is composed of nine generations (e.g., four generations of respiratory bronchioles, four generation of alveolar ducts, and one generation of alveolar sacs). This small tree of gas‐exchanging airways is called acinus and represents the functional unit of the lung.

Rats and other rodents possess rather a monopodial than a dichotomous branching pattern. The trachea divides into a left and a right main bronchus that ventilates the left and the right lung, respectively. Arising from the right main bronchi, lobar bronchi serve the right upper, the right middle, the right lower, and the cardiac lobe (Yeh et al. 1979). Secondary bronchi arise from these main lobar bronchi and ventilate the distal regions of the lobes over a system of smaller bronchi and bronchioles. In contrast to human lungs, rodent lungs do not contain respiratory bronchioles, but only one generation of a transitional bronchiole (Rodriguez et al. 1987). The latter one directly connects the conducting airways to the most proximal alveolar ducts. While the proximal part of transitional bronchiole is purely conducting, the first alveoli appearing in its distal part mark the beginning of the gas‐exchanging area. Rodriguez et al. defined “the complex of alveolated airways distal to the terminal bronchiole” as acinus.

The estimation of the number of acini in the lung was attempted several times using various techniques. Unfortunately, these methods were so time consuming that the number of analyzed samples was limited. Yeh et al. (1979) quantified the number of terminal bronchioles by counting them on a silicone rubber cast of a bronchial tree. The number of acini can be read out of those data by doubling the number of terminal bronchiole because the acinus is located one generation more distally. Mercer and Crapo obtained similar results by counting ventilatory units (airways distal of the bronchiole–alveolar duct junction) on serial sections of the right upper and lower lung lobe. As long as no respiratory bronchioles exist in a species (e.g., rats), the ventilatory units are identical with the acini which means that the sharp border between the conducting and gas‐exchanging airways lies inside the transitional bronchiole. It is identical with the entrance into the acini and with the bronchiole–alveolar duct junction. Rodriguez et al. (1987) attempted to use serial sectioning, but changed rapidly to using airways cast to study pulmonary acini. Serial sectioning is a precise method but demands slicing, imaging, and aligning of several thousand of slices for an entire lung lobe, which is too laborious even for a small number of samples. Thus, subvolumes of airways casts were used. Wulfsohn et al. (2010) proposed another approach. They defined an unbiased estimator based on counting the entrance of acini using a dissector of five consecutive sections. Complex sampling, as well as alignment of and counting on the five sections prevent studies using larger numbers of samples. More recently three‐dimensional (3D) imaging methods, using X‐ray computed tomography were used for studying lung morphometry. Sera et al. (2003) and Lee et al. (2008, 2011) scanned airway casts using μCT devices. Unfortunately, no statements on the number of acini were made and the used casting techniques often lead to incomplete filling of the airways causing a bias in the estimation method. Direct imaging of lung tissue was achieved by Vasilescu et al. using μCT and by Haberthür et al. (2013) using synchrotron based X‐ray tomographic microscopy. They focused on acinar morphometry and did not count the number of acini directly. They estimated the volume, surface area, or number of alveoli per acinus of a larger number of acini and used the means of these values to calculate the number of acini based on the total volume, surface area, or number of alveoli of the entire lung, respectively (Vasilescu et al. 2012; Haberthür et al. 2013). McDonough et al. (2011) used a multimodal approach (multidetector CT and μCT) to compare structural changes during different stages of chronic obstructive pulmonary disease (COPD) in humans. Specific regions of interest of the lungs were selected and scanned using a μCT. On the resulting datasets, the acinar density was counted and the total number of acini estimated. A decrease in the number of acini was reported for COPD patients. This method uses a sampling method that focuses on specific region of interest for COPD, but did not focus on the total number of acini.

In the presented study, we describe a method for an efficient estimation of the total number of acini. Based on Zeltner et al. (1990) and own pilot studies we observed that one lung lobe is the minimum appropriate subvolume for the estimation of the total number of acini. Thus, virtual serial sections of one entire lung lobe were gained by X‐ray computed microtomography (μCT) or synchrotron radiation‐based X‐ray tomographic microscopy (SRXTM), and analyzed manually. Reliable detection of acini was based on morphological criteria (wall thickness of bronchioles and appearance of alveoli). The obtained number of acini per lung lobe was used to calculate the mean acinar volume and the total number of acini per lung.

## Method

### Animals

The lungs of eight young adult male rats (day 60, Wistar Bern) were obtained and were prepared as described previously (Mund et al. 2008). Briefly, the air space was filled with 4% paraformaldehyde (PFA) in phosphate‐buffered saline (PBS) via tracheal instillation at a constant pressure of 20‐cm water column. The lungs were removed and the pressure was maintained during fixation for a minimum of 2 h at 4°C in order to prevent a recoiling of the lung (Luyet et al. 2002). After fixation, the lobes were separated and their volume was measured by water displacement (Scherle 1970). A second volume measurement was performed using the Cavalieri principle (Gundersen and Jensen 1987) on the tomographic dataset to determine the volume shrinkage of the samples.

Three right middle lung lobes (samples A–C) were prepared for synchrotron radiation based X‐ray tomographic microscopy (SRXTM) by postfixation with 1% osmium tetroxide and staining with 4% uranyl acetate to increase the X‐ray absorption contrast. Using Histoclear (Merck KGaA, Darmstadt, Germany) as an intermedium the samples were dehydrated in a graded series of ethanol and embedded in paraffin (Haberthür et al. 2010, 2013). The five remaining right middle lung lobes (samples D–H) were prepared for μCT scanning by critical point drying. Samples were dehydrated in a graded series of ethanol (70–100%) and dried at a pressure of 82 bar in CO_2_ (Kaeslin et al. 2005). All samples were mounted onto standard scanning electron microscopy sample holders (PLANO GmbH, Wetzlar, Germany).

The handling of animals before and during the experiments as well as the experiments themselves was approved and supervised by the Swiss Agency for Environment, Forest and Landscape and the Veterinary Service of the Canton of Bern, Switzerland.

### Synchrotron radiation‐based X‐ray tomographic microscopy

Tomographic imaging was performed at the TOMCAT beamline (Stampanoni et al. 1970) of the Swiss Light Source at the Paul Scherrer Institute in Villigen, Switzerland. The samples were scanned at an energy of 20 keV. X‐rays were converted to visible light by a scintillator (20‐*μ*m‐thick LuAG:Ce or 18–*μ*m‐thick YAG:Ce; Crytur Ltd., Turnov, Czech Republic) after crossing the samples. An optic microscope magnified the visible light to obtain an effective voxel side length of 2.35 *μ*m. In order to visualize the entire lung lobe, the field of view was increased perpendicular to the rotational axis by a factor of 3 using “wide‐field SRXTM” (Haberthür et al. 2010). In addition, 5–7 wide‐field scans were stacked in parallel to the rotational axis, resulting in eight bits grayscale 3D stacks of images of approximately 7500 × 7500 × 10000−15000 voxels.

### μCt

Samples (D–H) were imaged by a μCT device (SkyScan 1172; Bruker, Billerica, MA) at 33 kV and 204 *μ*A without filtering. Two to three scans in the vertical direction (oversize scan) were needed to visualize the entire sample at 2.52–3.45 *μ*m voxel side length. The GPU reconstruction software (NReconServer64bit; Bruker) was used on a GeForce GTX 680 (Nvidia corp., Santa Clara, CA) graphic card to reconstruct eight bits grayscale image stacks of approximately 4000 × 4000 × 5000−8200 voxels.

### Detection and counting of acini

**Figure 1.**
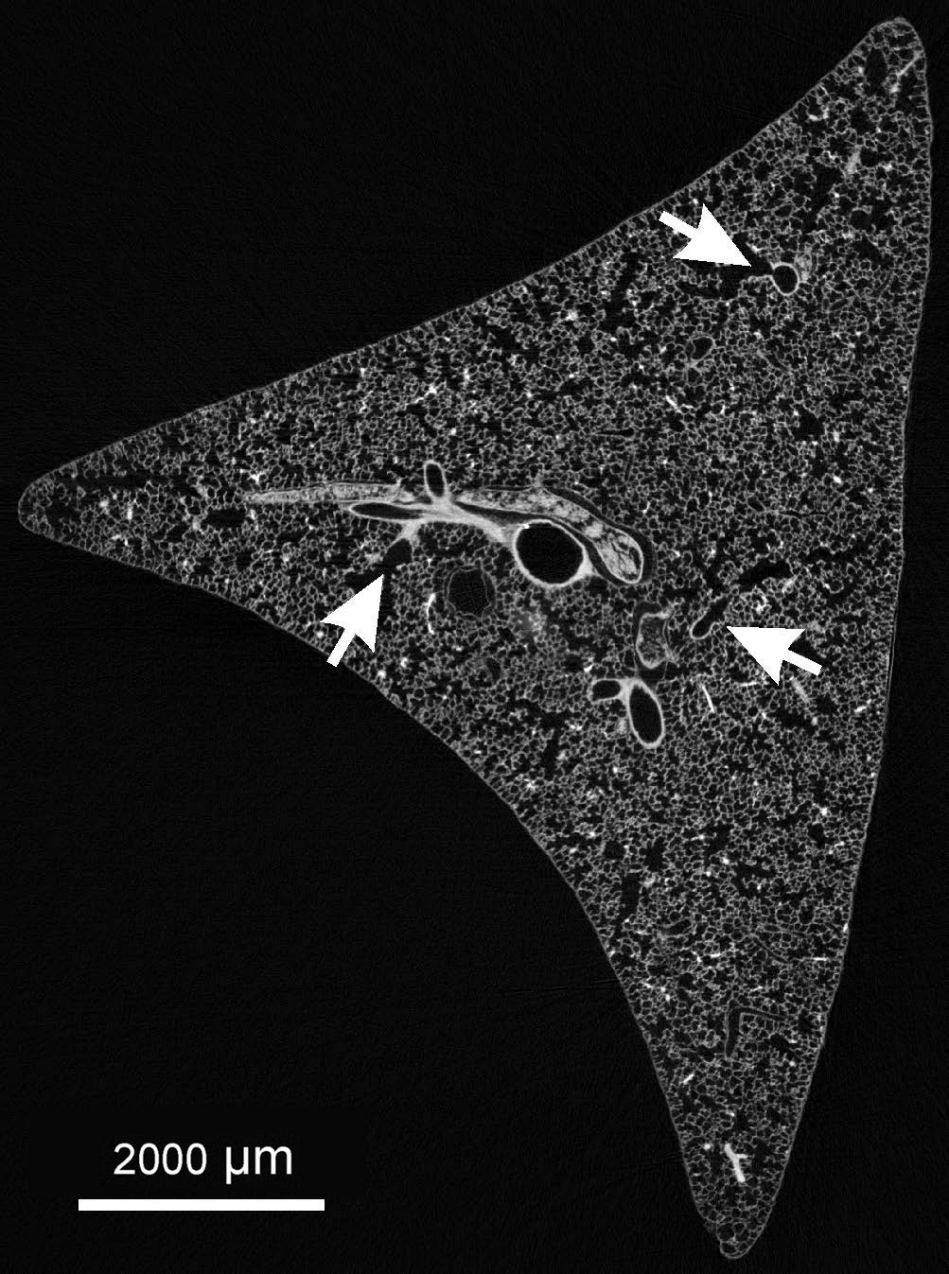
Cross section of a right middle rat lung lobe. The arrows point to acinar entrances as they are recognized in one section. Morphological criteria (thickness of the wall of the airways and appearance of alveoli) were used to detect the transition from purely conducting to gas‐exchanging airways. This transition represents the entrance of an acinus.

Stack of virtual serial sections of eight right middle rat lung lobes were taken by SRXTM or μCT at a voxel side length of 2.35–3.45 *μ*m. In order to reduce the computing power requirements, stacks were subdivided into 250–500 images using Fiji (Schindelin et al. 2012). By scrolling through those substacks, the acini entrances (Fig. 1) were detected as described in the result section, labeled manually, and counted using a laboratory counter (Clay Adams, New York, NY). After one substack was counted, labels of overlapping acini were reported manually to the next substack to exclude double counting.

### Determination of the minimal required resolution of images

Four regions of interest containing 13, 39, 34, or 25 acini were selected. The subvolumes, originally imaged at a voxel side length of 2.35, 2.35, 2.52, and 3.25 *μ*m, respectively, were resized using bi‐cubic interpolation to simulate different voxel side lengths (3, 3.5, 3.6, 4, 5, and 10 *μ*m). Afterward the number of acini was counted following the above‐described procedure. The limit of the required resolution for the analysis was set to one step better than the resolutions where the number of acini started to decrease.

### Visualization

*3D visualizations of the lung parenchyma*. Volume of interest was selected and displayed by the standard volume rendering module (View3D) of MeVisLab (version 2.1, 2010‐07‐26 Release; MeVis Medical Solutions and Frauenhofer MEVIS‐Institute for Medical Image Computing, Bremen, Germany).

In order to visualize the conducting airways in one right middle lung lobe, the entrance of the acini was labeled and closed with segmentation stoppers using a custom‐made MeVisLab pipeline (Haberthür et al. 2013). Briefly, 15 stacks of 250 images were postproceeded using MeVisLab. The substacks were loaded as TIFF files and segmentation stoppers were set manually at the acinar entrances in order to separate the acini from the conducting airways. The conducting airways were segmented using a gray‐level threshold‐based region‐growing algorithm (Zucker 1976) after down‐sampling by a factor of 2. The segmentation seed points were set manually within the conducting airways. All 15 segmentations were reconstructed as one three‐dimensional model using a custom‐made MeVisLab pipeline. This basic pipeline stacked all segmentations of the 15 substacks and displayed spheres at the segmentation stopper positions. The segmentation and all other computer works were performed on a Dell (Round Rock, TX) Precision T5500 work station (Intel Xeon X5650 (six Core, 2.67 GHz), 24 GB RAM, Windows 7 Professional 64).

### Statistics

The statistical analyses used Microsoft Excel (version 14.0.7106.5003, 32‐bit) and Prism (version 5.04; GraphPad Software Inc., La Jolla, CA). The values are expressed as means (±standard deviation). The coefficient of variation (CV = standard deviation/mean) was used to compare deviations of various parameters under normalized conditions. *T*‐tests and analysis of variance (ANOVA) were used to analyze the statistical significance of difference for two or more groups, respectively. A significance level *α *= 0.05 was used for both test procedures. Regression analysis was used to test for linear combination of two parameters (e.g., number of acini and lung volume). The linear regression of the observed values and the *R*^2^ (coefficient of determination) were calculated. This coefficient indicates how well the observed values fit the “ideal” values of a linear regression. In this case, *R*^2^ is the square of the Pearson correlation coefficient (*r*). No correlation between parameters was assumed when *R*^2^ ≤ 0.5 (|*r*| ≤ 0.7), weak correlation when 0.5 < *R*^2^ ≤ 0.7 (0.7 < |*r*| ≤ 0.84), and strong correlation when 0.7 < *R*^2^ (0.84 < |*r*|).

### Calculations

The acinar airspace volume was calculated by dividing the lobe volume by the counted number of acini and multiplying it by 0.658 (volume density of the airspace of the parenchyma). Similarly, the mean acinar volume (tissue plus airspace of an acinus) was calculated by multiplying by 0.888, 0.839, 0.843, 0.865, and 0.862 (volume density of parenchyma) for right upper lobe, right middle lobe, right lower lobe, cardiac lobe, and left lung, respectively. All densities were determined by point counting following the ATS guidelines (Hsia et al. 2010). The total number of acini per lung was calculated by dividing the counted number of acini per lung lobe by the parenchymal volume of the lung lobe and multiplying it by the total parenchymal lung volume. All volumes used for the calculations were determined by water displacement in order to avoid a systematic error due to shrinking of the tissue.

## Results

### Detection of acini

**Figure 2.**
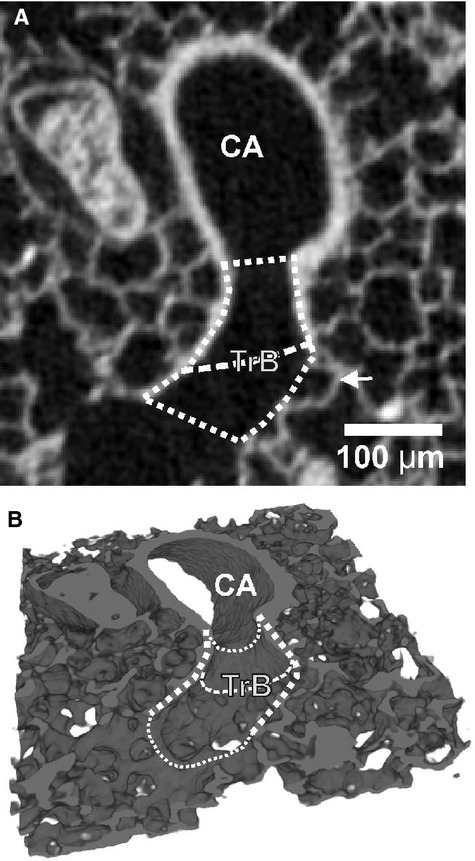
Structural alterations at the entrance of an acinus. Every acinus is ventilated by its transitional bronchiole (TrB, dotted line), which itself is ventilated by a purely conducting airway (CA). The conducting airway may be a terminal or larger bronchiole. Its inner surface is covered with cuboidal epithelium which looks like cobble stone pavement in this kind of reconstructions. The entrance of the acinus (dashed line) is located at the sudden transition of the thick wall typical for a conducting airway to the thin wall typical for the gas‐exchange area. The most proximal alveoli (arrow) appear directly distal of this transition. Dotted lines, wall of the transitional bronchiole.

**Figure 3.**
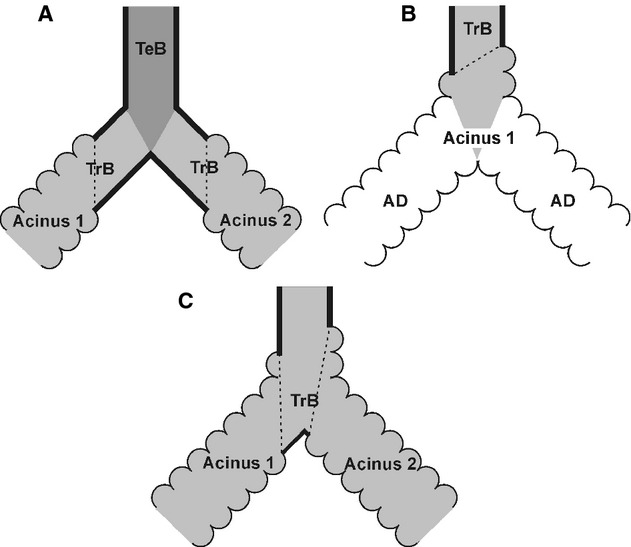
Schematic drawing comparing unbranched and branched transitional bronchioles. Typically the last branching point before the entrance of an acinus is located in the purely conducting part of the bronchial tree (A). In this case, the next branching point is located inside the acinus and represents a branching of alveolar ducts (B). However, relative frequently a branching point is located in the zone of transition from the conducting to the gas‐exchanging airways (C). Hence, the branching point itself shows still the thick wall of the conducting airways, but alveolated airway walls were observed proximal and distal of the branching point. Dark gray, terminal bronchiole (TeB); light gray, transitional bronchiole (TrB); white, alveolar duct (AD); dotted line, transition from the conducting to the gas‐exchanging airways.

Wulfsohn et al. (2010) developed a method where they used a dissector of five consecutive sections to estimate the number of acini. However, the effort necessary to cut, stain, align, and analyze hundreds of sections appeared too much for our goal to estimate the number of acini in large number of lungs (20–30). Based on the idea of Wulfsohn et al. and of McDonough et al. (2011), we used X‐ray tomographic datasets and developed a protocol to count pulmonary acini on intrinsically aligned stacks of tomographic images. On virtual section (Fig. 1), we were able to recognize the entrance of the acini by scrolling through a 3D stack of images. In rat lungs, the entrance of the acinus represents the quite sudden transition from a purely conducting to gas‐exchanging airway. Per definition it is localized in the transitional bronchiole (Rodriguez et al. 1987). We used this sudden change of the thickness of the wall from the conducting part to the gas‐exchanging part of the transitional bronchiole, as well as the appearance of alveoli in the wall of the airways as morphological criteria for the detection of the acini (Figs. 2, 3).

**Figure 4.**
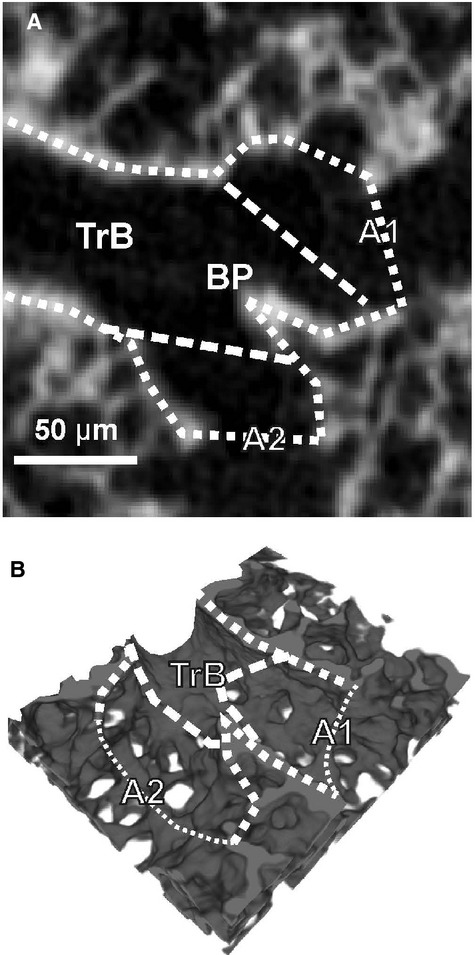
Dual acinar entrance in one transitional bronchiole. If the transitional bronchiole (TrB) contains a branching point (BP), two acini (A1, A2) share the same transitional bronchiole. The shown branching point is still inside the transitional bronchiole and not part of one larger acinus, because at the branching point itself the wall of the airway is still thick and does not contain alveoli. Dashed line, transition conducting airways–gas‐exchange area; dotted lines, wall of the transitional bronchiole.

Typically we did not observe any branching point in the transitional bronchiole (Figs. 2, 3A and B). However, the border between the conducting and the gas‐exchanging surface area does rarely form a circle perpendicular to the axis of the transitional bronchiole. It is better represented by an ellipse in which plane forms an angle to the axis of the airway. As a result the transition from the bronchiolar epithelium (cuboidal epithelium) to the wall of the alveolar ducts (basically the entrance rings of the alveoli) takes place in a small part of the transitional bronchioles, where both wall structures are located in the same small segment. Relative frequently a branching point of the airway lays directly in this small segment of a transitional bronchiole. In this case, we observed the typical thick wall structure of the conducting airways at the branching point itself (Figs. 3C, 4). Because the beginning of the gas‐exchange area defines the entrance of an acinus, we counted the acini of these transitional bronchioles as two acini (Figs. 3C, 4).

### 3D Visualization of the conducting airways

**Figure 5.**
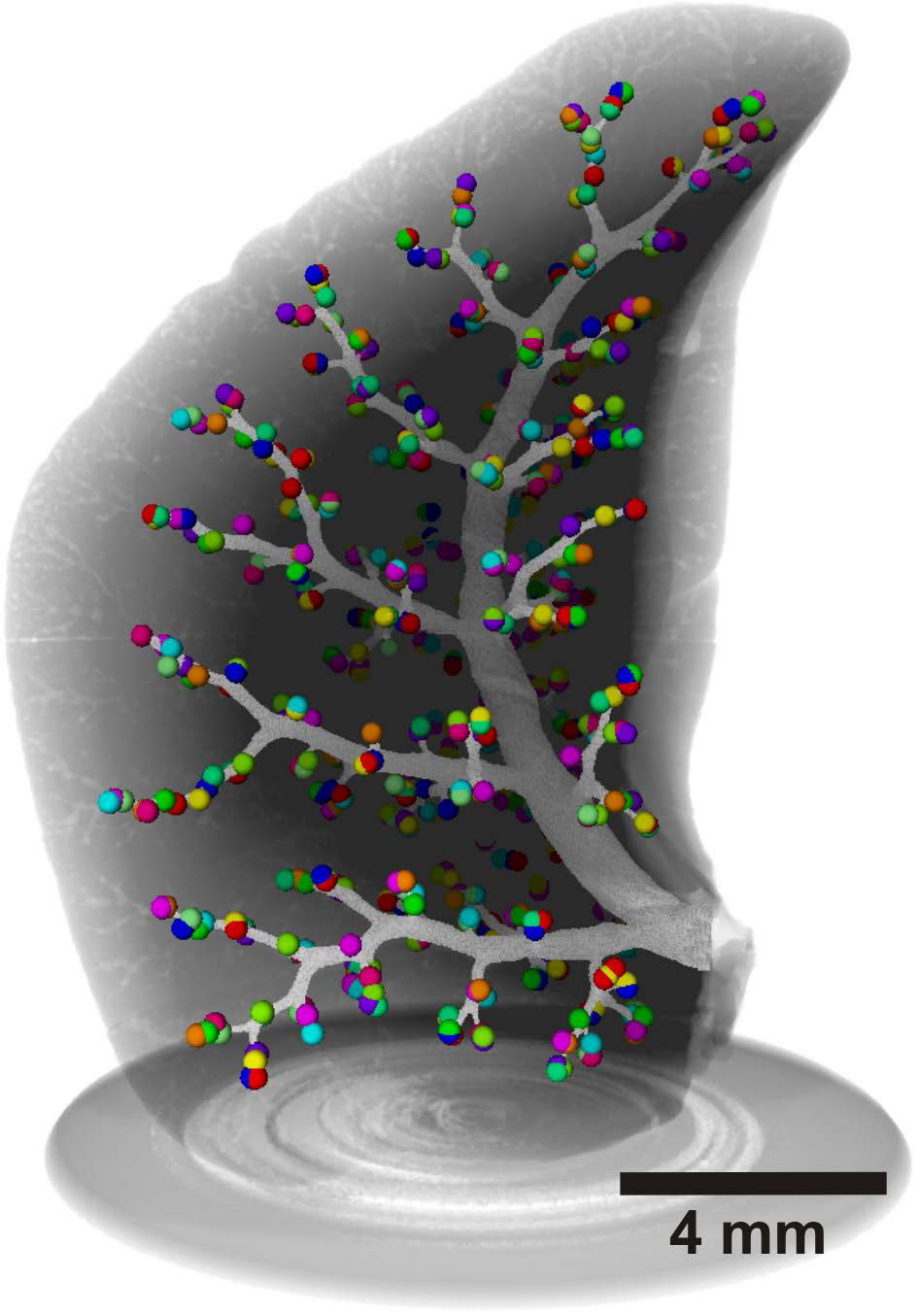
Tree of conducting airways shown inside a right middle lobe (young adult rat). The walls of the conducting airways are shown in light gray and the spheres represent the acini entrances. The gray shadows delimit the lobe tissue and the sample holder. This 3D reconstruction illustrates that the bronchial tree of rat does not follow the dichotomous branching pattern of the human lung.

**Figure 6.**
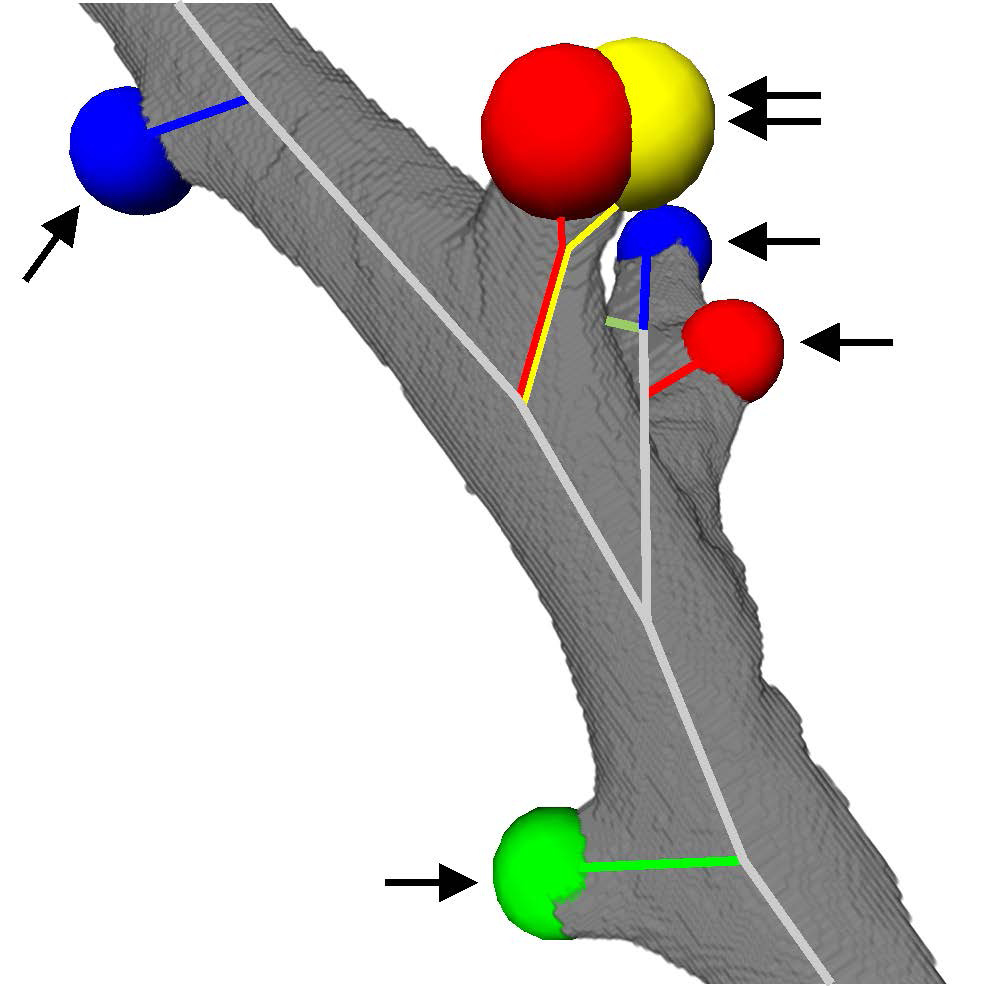
3D visualization of the location of acinar entrances. A small part of a secondary bronchus (in gray) and the acinar entrances are shown (colored balls). The skeletons of the conducting airways are drawn in gray, the ones of the transitional bronchioles in color. Arrow, single entrance to an acinus; double‐arrow, two acinar entrances very close together as shown in Figures 3C, 4.

In order to study the global arrangement of the acinar entrances, we visualized the conducting airways of one middle right lung lobe by closing off the acini at their entrance using segmentation stoppers and applying a region‐growing algorithm (Fig. 5). The 3D‐visualization illustrates the monopodial branching pattern of the conducting airways, including one main lobar bronchus and secondary lobar bronchi pointing to the three edges of the middle right lobe. These secondary lobar bronchi ventilate bronchi/bronchioles that are connected to the acini by short transitional bronchioles. Most of the transitional bronchioles and the acinar entrances (Fig. 5, colored spheres) were observed in a cylinder around the lobar secondary and tertiary bronchi. As the outermost layer of the lung parenchyma, the cortex does not contain any conducting airways. It possesses acinar airways only. The acinar entrances itself were detected at various generations of the airway tree. The most proximal acini appeared already at intralobar generation 4 while distal acini were located at intralobar generation 15 and higher. Many transitional bronchioles arise directly from secondary or tertiary bronchi (Figs. 3, 4, 6). Typically each bronchiole ends in only one acinus, but relative frequently we observed two acini located very close to each other (Fig. 6). The latter represent a branching point inside a transitional bronchiole (Figs. 3, 4).

### Counting of acini

The right middle lung lobes of eight young adult rats (day 60, Table 1) were imaged either by synchrotron radiation‐based X‐ray tomographic microscopy (SRXTM) or by μCT and sampled. Different sampling methods (as defined in the ATS guidelines (Hsia et al. 2010)) were tested to estimate the number of acini. None of them delivered accurate results even at large sampling rates (50% of a lobe). This is explained by an inhomogeneous distribution of the acini within a lobe (Fig. 5). However, one lobe still represents a representative fraction of the entire lung (Zeltner et al. 1990; see below). We decided to count the entire lobe volume, because at the end the sampling procedure did not save any labor.

**Table 1.** Basic parameter of the animals studied.

Sample	Body weight	Lung volume	Lobe volume								
			Right lung								LL
			RUL	RML					RLL	LC	LL
				Water displacement	Cavalieri		Shrinkage				
	[g]	[mL]	[mL]	[mL]	[mL]		[%]		[mL]	[mL]	[mL]
A	291.00	7.03	0.727	0.824	0.591^1^		28.23^1^		2.02	0.862	2.59
B	315.20	7.43	0.767	0.836	0.590^1^		29.40^1^		2.24	0.860	2.73
C	306.80	7.44	0.737	1.04	0.747^1^		28.47^1^		2.07	0.989	2.60
D	280.95	7.02	0.740	0.985	0.388^2^		60.61^2^		1.97	0.847	2.49
E	316.25	8.07	0.997	1.04	0.393^2^		62.19^2^		2.14	1.07	2.83
F	294.93	6.82	0.731	0.80	0.316^2^		60.53^2^		1.95	0.912	2.43
G	294.05	8.15	0.903	0.924	0.348^2^		62.34^2^		2.43	1.01	2.87
H	267.41	8.14	0.964	1.05	0.377^2^		64.20^2^		2.30	1.09	2.73
Mean	295.82	7.51	0.821	0.938	0.643^1^	0.364^2^	28.70^1^	61.97^2^	2.14	0.954	2.66

RUL, right upper lobe; RML, right middle lobe; RLL, right lower lobe; LC, cardiac lobe; LL, left lung.

^1^ Paraffin‐embedded samples.

^2^ Critical point dried samples. Shrinkage was defined as the volume difference (in percent) of the samples before embedding (water displacement) and after scanning (cavalieri).

### Number of acini

We counted a mean of 686 (±89) acini per right middle lobe and calculated a mean acinar volume of 1.16 (±0.138) *μ*L and a mean acinar airspace volume of 0.907 (±0.108) *μ*L. Based on these numbers we estimated that a young adult rat lung contains 5612 (±547) acini (Table 2). This was estimated for eight rats. In addition, we calculated the mean number of acini for the four remaining lung lobes based on the exhaustive counting of these 12 lobes (*N* = 3 per lobe, Table 2).

**Table 2.** Validation that the right middle lobe represents a valid sample for estimation of the number of acini in the entire lung and individual lung lobes.

	Lobe variability			Estimation method				
	Mean acinar volume	Difference to RML		Counted	Estimated	Counted vs. Estimated		Matching accuracy
	[*μ*L]	*t*‐test	Significant?	[# Acini]	[# Acini]	*t*‐test	Significant?	[%]
RUL	1.06 ± 0.018	0.109	No	689 ± 138	668 ± 126	0.144	No	97.09 ± 1.84
RML	1.16 ± 0.138			686 ± 89				
RLL	0.96 ± 0.066	0.117	No	1808 ± 103	1689 ± 77	0.201	No	93.59 ± 5.64
LC	1.24 ± 0.123	0.211	No	734 ± 66	780 ± 80	0.215	No	106.33 ± 6.36
LL	1.22 ± 0.095	0.278	No	1973 ± 192	2095 ± 153	0.275	No	106.49 ± 7.89
Entire Lung	1.11 ± 0.044	0.978	No	5943 ± 521	5971 ± 388	0.869	No	100.80 ± 5.16

Based on the acinar counting of eight right middle lobes (rats day 60) we estimated the number of acini in the remaining lung lobes by dividing their parenchymal volume by the mean acinar volume of the right middle lobe. To validate the estimation we counted the acini of the remaining four lobes (*N* = 3) and did not observe any significant difference. RUL, right upper lobe; RML, right middle lobe; RLL, right lower lobe; LC, cardiac lobe; LL, left lung. Matching accuracy is the estimation divided by counted number of acini expressed in percent.

The number of acini for the remaining lobes was estimated based on the volume fraction of parenchyma and the counted number of acini in the right middle lung lobe. In order to verify these estimations the acini of the four remaining lungs lobes were also counted (*N* per lobe = 3, Table 2). On average, our estimated values were 0.88% higher than the counted values. Estimations for the entire lung represented 100.80 (±5.16) % of the counted values and the largest mean absolute difference per lobe was 6.49 (±7.89) % (left lung). No significant differences between counted and estimated number of acini were found in any of the lobes (Table 2). Therefore, the right middle lobe represents a valid sample for the entire lung and any other lobe. However, this is not necessarily true for every lobe. Therefore, we tested if the mean acinar volume varies between different lung lobes.

### Mean acinar volume

We calculated the mean acinar volume of all lobes based on counted number of acini and the parenchymal volume of each lobe (Table 2). Applying the ANOVA *F*‐test a significant difference (*P* = 0.0395, *α *= 0.05) for the mean acinar volumes was observed. In addition, the ANOVA tests showed that the mean acinar volume of the right lower lobe is significantly different to the mean acinar volume of the four other lobes. Therefore, the right lower lobe should not be used as sample for the other lobes.

### Correlation among lung volume, body weight, and number of acini

**Figure 7.**
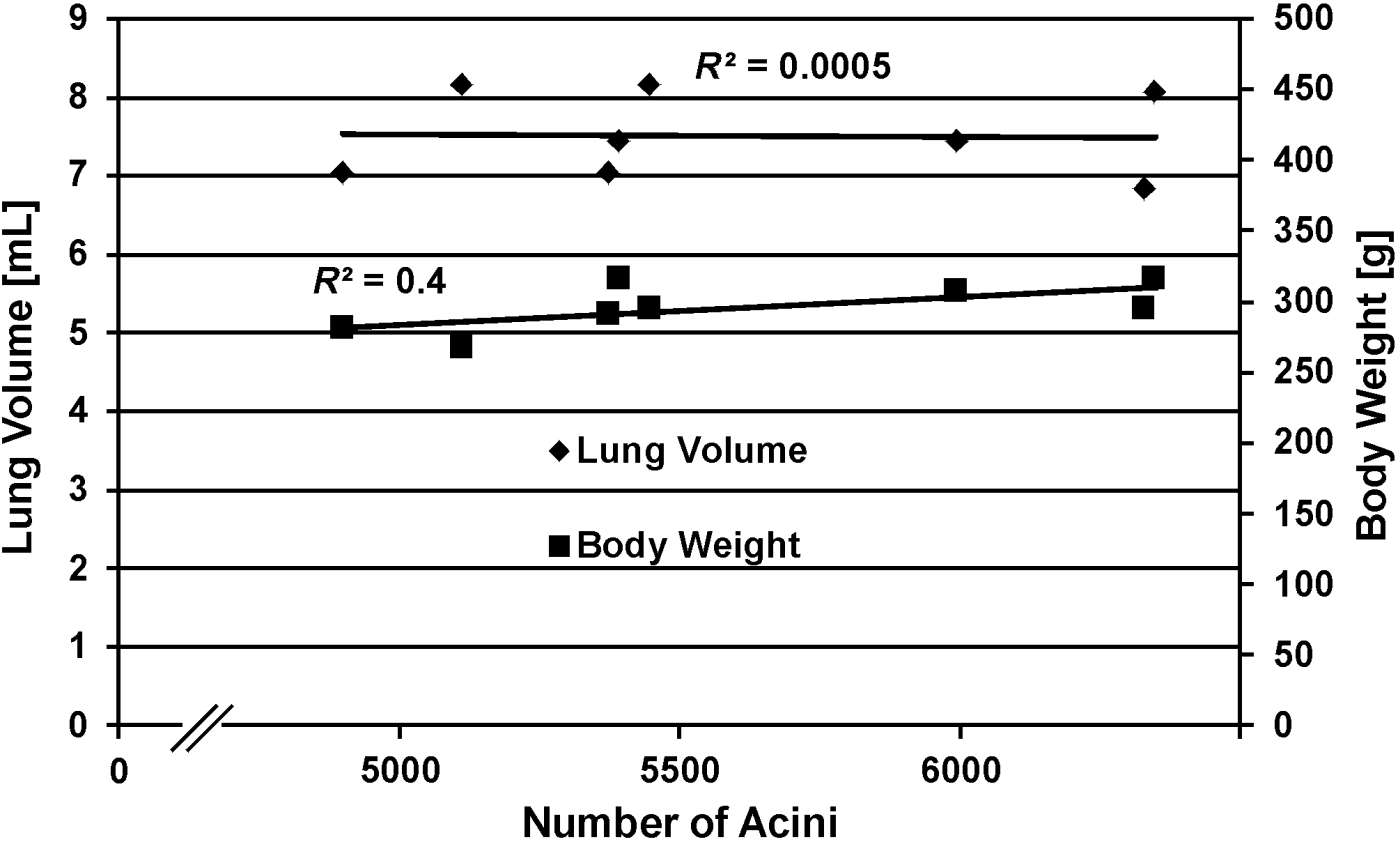
Correlation among lung volume, body weight, and number of acini at postnatal day 60. The coefficient of determination (*R*^2^) was calculated to analyze the correlation between different lung parameters. 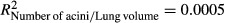 and 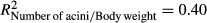. These coefficients indicate that the number of acini (mean: 5612) is independent of the lung volume and the body weight at postnatal day 60 of rat lung development.

The coefficient of determination (*R*^2^) was used to describe the correlation between base parameters (i.e., lung volume, body weight) and the number of acini. At day 60, no correlation was observed between any of the four parameters tested (lung volume, body weight, mean acinar volume, and number of acini, Fig. 7).

### Required resolution of images and shrinking of the tissue

The resolution – or the ability to identify any (morphological) structure on images – is directly dependent on the size of the pixel (2D images), or voxel (3D images), respectively. A pixel/voxel should be significantly smaller than the smallest feature of interest (Sterio 1984). Thus, the side length of the isometric voxel had to be shorter than the smallest acinus entrance and/or the thickness of the wall of the terminal bronchioles. Regions of interest containing acini of various sizes were analyzed at different isometric voxel side lengths. Between 2.35 and 3.6 *μ*m voxel size the counting of acini gave exactly the same results independently of the voxel size. At a voxel side length of 4 *μ*m and larger the number of counted acini started to decrease. Therefore, we defined a voxel size of 3.6 *μ*m or smaller to be necessary for correctly assessing the number of lung acini.

3D imaging of the first three samples were done by synchrotron radiation‐based X‐ray tomographic microscopy (SRXTM) at a voxel size of 2.35 *μ*m. Due to a limitation of available beam time the remaining five samples were scanned using a high‐resolution μCT at a voxel size of 2.52–3.45 *μ*m. As expected, the image quality was better on SRXTM‐images than on μCT images. Nevertheless, the resulting resolution of both methods was detailed enough for the counting of the acini. Because the energy levels of μCT and SRXTM were different, we used unstained critical point dried lungs in the μCT instead of heavy metal stained and paraffin‐embedded lungs as used for SRXTM. Otherwise the X‐rays of the μCT would not have penetrated the sample. We determined the shrinkage of the tissue after both embedding procedures. The volume decreased to 62.0 (±1.5) % after critical point drying and to 28.70 (±0.62) % after paraffin embedding. Because we counted every acinus in one lung lobe, the shrinkage did not have any influence on the number of acini.

## Discussion

An acinus is defined as the complex of alveolated gas‐exchanging airways distal of one terminal bronchiole (most distal still purely conducting bronchiole). It represents the functional unit of the lung parenchyma. In humans an acinus starts with four generations of respiratory bronchioles (partly conducting and partly gas‐exchanging bronchioles) followed by four generations of alveolar ducts and the sacculi (Weibel 1963). Rats and mice do not possess respiratory bronchioles. The transition from the conducting airways to the alveolar ducts takes place in one generation of transitional bronchioles. A rat acinus contains an average of six generations (4–10 generations) of airways and has a mean volume of ~2 *μ*L (range 0.5–5 *μ*L) (Rodriguez et al. 1987).

### Estimation of the number of acini

Several approaches to estimate the number of acini were developed during the last three to four decades. They are based (1) on physical serial sections (Mercer and Crapo 1987; Wulfsohn et al. 2010); (2) on casts of the bronchial tree; and/or (3) on the estimation of the acinar volume or the number of alveoli per acinus using X‐ray tomographic datasets (McDonough et al. 2011; Vasilescu et al. 2012; Haberthür et al. 2013; for additional detail see Introduction). Unfortunately, all of these methods require a tremendous amount of labor. This is the reason that so far only studies with very small numbers of samples were performed. Only McDonough et al. (2011) presented a method permitting to analyze a large number of samples. The sampling used in their method was not applicable to rat lung due to the highly inhomogeneous distribution of acini within one lobe. We thus developed another sampling procedure to efficiently count acini in rat lungs. Using X‐ray tomographic datasets (virtual serial sections) obtained by synchrotron radiation‐based X‐ray tomographic microscopy or microcomputed tomography, the number of acini for eight middle lung lobes of young adult rats (day 60) were counted. While scrolling through the stack of virtual sections we recognized the acinar entrance by the sudden change of the thickness of the wall from the conducting part to the gas‐exchanging part of the transitional bronchiole, as well as the appearance of most proximal alveoli. Due to the very uneven distribution of the acinar entrances, the required sampling volume exceeded 50% of the lung lobe (Fig. 5). Therefore, we omitted any sampling and counted the acini of an entire lung lobe. This was much faster than counting only parts of the lobe. As a result we established a method for an unbiased, efficient, and reliable counting of acini which now may be applied to large numbers of samples.

### Number of acini

We estimated that a young adult rat lung possesses approximately 5600 acini (Table 2, mean of eight animals). Rodriguez et al. (1987) estimated 4020 and Haberthür et al. (2013) 5470 acini per rat lung. Mercer and Crapo (1987) and Yeh et al. (1979) estimated the number of terminal bronchioles in rat lungs (2020 or 2490, respectively). Because a terminal bronchiole ventilates two acini (Rodriguez et al. 1987), these numbers correspond to 4040 and 4980 acini per rat lung. Our number is 10–30% higher than the other ones. It may be explained by the following reasons.

First, we observed between ~4900 and ~6300 acini per rat lung. In addition, we have to expect variation due to the used strain (Osmanagic et al. 2010) and due to the small numbers of animals studied.

Second, we encounter an effect which is partly similar to the coastline paradox where a measurement of a length of a coastline depends on the scale or resolution of the measurement (Mandelbrot 1967). Frequently, the transitional bronchiole contains a branching point very close to the entrance of two acini. In this case, the wall thickness represented our main criteria to distinguish between one versus two acini (Figs. 3, 4). However, only a small part of the “thick” wall located in the small angle between the two daughter branches made the difference (Figs. 2–6). In casts of the airways (Yeh et al. 1979; Rodriguez et al. 1987), where the wall thickness may not be judged and at lower resolution such a small piece of “thick” wall cannot be recognized. This leads to a systematic underestimation of the number of acini. We determined that a minimal resolution of 3.6 *μ*m isometric voxel side length is sufficient for the reliable counting of rat acini. However, counting different species may require a different resolution.

Third, Mercer and Crapo bypassed these problems by counting acini on serial sections. Due to the tedious work required for slicing and aligning of the sections, only four subsamples of 4 × 4 × 4 mm^3^ of the right upper and lower lobe were analyzed. As shown in Figure 5 acinar entrances are located on the surface of a cylinder surrounding the bronchi and bronchioles. Furthermore, a cortex which is free of conducting airways enwraps the lung lobe. Thus, the uneven distribution requires adapted sampling protocols where the sampled fraction and the number of sampled subregions will be very high. We basically repeated Mercer's and Crapo's experiments using virtual X‐ray tomographic sections. Using this kind of section we were able to save all the labor required for sectioning and alignment of the section. At least in our hands four samples of 4 × 4 × 4 mm^3^ resulted in an estimation error which may explain the difference between Mercer and Crapo's data and ours.

### Coefficient of variation

As a mean of eight lungs we estimated 5612 (±547) acini and a coefficient of variation of 0.097. The coefficient of variation represents the standard deviation divided by the mean. This deviation is the summation of different errors (e.g., counting error, systematic error of the method, and the individual variations). The deviation of our method was in the same ranges as the individual variations of the lung volume (7.51 mL CV 0.072) and body weight (295.82 g CV 0.057). Unfortunately, most of the previous studies did not investigate the variations. The accurate method from Wulfsohn et al. obtained a mean of 4484 acini (CV 0.06) for adult (day 69) female mice and 4199 acini (CV 0.11) for 21‐day‐old mice. The coefficients of variation are in the same range even if a limited number of mice were used (three for day 21 and two for day 69). Haberthür et al. (Haberthür et al. 2013) indirectly estimated 5470 acini (CV 0.15) on three adult rats, which is close to our results. McDonough et al. (2011) estimated human lung and thus we could not compared the total number of acini. However, they obtained similar coefficient of variation (CV 0.17) for healthy patients.

### Mean acinar volume

Based on the total lung volume and the number of acini we calculated a mean acinar airspace volume of 0.907 (±0.108) *μ*L, which is similar to previously published data. Haberthür et al. (2013) estimated 1.148 (±0.322) *μ*L and Mercer and Crapo 1.044 (±0.112) *μ*L per acini (0.522 (±0.056) *μ*L per ventilatory unit). However, Rodriguez et al. reported twice as large acinar volume (2.17 *μ*L). Comparing these rat data with mouse data, the variation of the rat data appears to be relative small, because in mice up to fourfold of the lung volumes have been observed in different studies or strains.

### Lobe variability

The number of acini for the entire lung and for the remaining four lobes was estimated based on the counting of all acini of the right middle lobe. To do so a constant mean acinar volume between right middle lobe and the other lobes was assumed. *T*‐tests did not reveal any significant differences in comparison to the right middle lobe (Table 2).

The assumption of constant mean acinar volume for the entire lung is not valid, because the right lower lobe showed a significant lower mean acinar volume than the other lobes. The reason of this interlobar variability was not assessed in this article but could arise due to biological variability or to inhomogeneous filling during instillation.

The relevance of these small differences remains an open question; on one hand they had very limited influence on the estimation of the number of acini. On the other hand these small differences will not substantially influence the physiology of an individual. The total number of acini is certainly of larger physiological relevance.

Since the mean acinar volume of the right middle lobe is not significantly different to any other lobe, we conclude that the right middle lobe is a valid estimator for the entire lung and the remaining lobes.

### Estimation

This method presented high accuracy (Table 2) for the estimation of the total number of acini (100.80%) and for all remaining lobes (100.88%). No significant differences were found between estimations and counted values. The mean absolute difference over all lobes was 5.53 (±5.25) %. Taking into account that the estimation of a single lobe is influenced by interindividual variability, counting error, and errors in volume measurement, this mean absolute difference is in an acceptable range. In addition, the absolute mean per lobe presented a maximal difference of ~6.5 (±7.9) %. In our opinion, this difference is a range which is expected for such an estimation method. The counted number of acini for right middle lobe varied from 594 acini to 824 acini. This corresponds to an absolute mean difference of 10.7 (±6.1) %. This showed that the estimation error is smaller than the interindividual variability.

### Correlation among lung volume, body weight, number of acini, and mean acinar volume

For a comparison of the above‐mentioned parameters, the coefficient of determination (*R*^2^) was used. No correlations between number of acini and lung volume (*R*^2^: 0.0005) nor body weight (*R*^2^: 0.4) were detected (Fig. 7). Furthermore, the number of acini did not correlate with the right middle lobe volume (*R*^2^: 0.26) or with the volume fraction (*R*^2^: 0.4) of this lobe. We conclude that the number of acini is independent of any of the mentioned parameters. The results also showed that for a specific age (postnatal day 60) the body weight and the lung volume are not correlating (*R*^2^: 27 × 10^−6^). However, because a correlation between body weight and lung volume exists throughout lung development (for our rat strain (Tschanz et al. 2003)), a similar correlation may or may not exist for the mean acinar volume.

### Synchrotron radiation‐based X‐ray tomographic microscopy (SRXTM) versus μCT

Two different scanning techniques were successfully applied in this study. Synchrotron radiation‐based X‐ray tomographic microscopy (beamline TOMCAT) was used as a gold standard. This is a fast and powerful technique but its accessibility is limited. To operate and maintain a μCT requires less effort, but even at the same voxel size the resolution does not match the one of SRXTM (monochrome vs. regular intensity white light, parallel vs. cone‐shaped beam). In our hands the counting of the acinar entrances was not affected by a lower quality of the μCT images, as long as the isometric voxel size was 3.6 *μ*m or smaller. However, for a segmentation of the alveolar ducts the application of SRXTM is necessary (Haberthür et al. 2013). Furthermore, μCT required critical point drying of the sample instead of paraffin embedding, because the X‐ray transmission of the heavy metal stained paraffin‐embedded samples was too low.

*In summary*, we present an efficient unbiased and robust method for the counting of acini. Avoiding cutting of physical sections, silicon casts of the airways, and sophisticated sampling protocols, we focus on efficient counting of an entire lung lobe. As a result, the presented method requires significant less effort and may be widely used as long a μCT is applied. It now enables us to count the number of acini even in a larger number of sample like in developmental studies or investigations of transgenic animals.

## Acknowledgments

We thank R. Mokso, G. Lovric, B. Pinzer, and F. Marone for their continuous support at the TOMCAT beamline and Eveline Yao and Mohammed Ouanella for expert technical assistance.

## Conflict of Interest

None declared.
